# Real-World Prevalence and Structural Validation of the Canonical 9p21 MTAP–CDKN2A/B Deletion in Non-NSCLC Solid Tumors

**DOI:** 10.3390/cancers18060893

**Published:** 2026-03-10

**Authors:** Miran Han, Eunbyeol Lee, Ji Eun Shin, Minsuk Kwon, Jung Yong Hong, Seung Tae Kim, Soomin Ahn, Kyoung-Mee Kim, Jeeyun Lee, Sung Hee Lim

**Affiliations:** 1 Division of Hematology-Oncology, Department of Medicine, Samsung Medical Center, Sungkyunkwan University School of Medicine, Seoul 06351, Republic of Korea; milanhan200@gmail.com (M.H.);; 2 Biointelligence Center, Samsung Precision Genome Medicine Institute, Samsung Medical Center, Seoul 06351, Republic of Korea; ebyeol44.lee@sbri.co.kr; 3 Department of Health Sciences and Technology, Samsung Advanced Institute for Health Sciences and Technology (SAIHST), Sungkyunkwan University, Seoul 06355, Republic of Korea; 4 Department of Pathology and Translational Genomics, Samsung Medical Center, Sungkyunkwan University School of Medicine, Seoul 06351, Republic of Korea

**Keywords:** MTAP deletion, 9p21.3 co-deletion, PRMT5 inhibitor, real-world data

## Abstract

**Background**: Deletion of the *MTAP* gene at chromosome 9p21.3 defines a therapeutically actionable molecular subset of cancers due to synthetic lethal vulnerability to PRMT5 and MAT2A inhibition. The real-world prevalence and genomic context of *MTAP* deletion in diverse solid tumors remain incompletely characterized. **Methods**: We retrospectively analyzed 579 solid tumor specimens subjected to next-generation sequencing-based copy-number profiling. The prevalence of *MTAP* deletion and its co-occurrence with *CDKN2A* and *CDKN2B* were evaluated, and genomic deletion patterns across chromosome 9 were systematically assessed. **Results**: MTAP deletion was detected in 14 cases (2.4%, 95% confidence interval [CI], 1.45–4.02%), with enrichment in sarcoma, pancreatic cancer, and urothelial carcinoma. Concurrent *CDKN2A* loss was observed in 92.9% of *MTAP*-deleted tumors, and 64.3% showed additional *CDKN2B* loss, indicating a coordinated focal deletion event at 9p21.3. Statistical analyses confirmed strong genomic associations between *MTAP* and neighboring tumor suppressor genes. Across the full cohort, deletion frequency peaked at the 9p21 locus, and among *MTAP*-deleted tumors, co-deletion frequency decreased with increasing genomic distance. All *MTAP*-deleted tumors were microsatellite stable and low tumor mutational burden (TMB-low). **Conclusions**: Our findings demonstrate that *MTAP* deletion is an infrequent but genomically coherent event in solid tumors, characterized by a canonical 9p21 co-deletion pattern. This real-world analysis underscores the importance of comprehensive genomic profiling to identify patients who may benefit from emerging *MTAP*-directed therapies.

## Simple Summary

Deletion of the *MTAP* gene is a genomic alteration of growing clinical interest because new targeted therapies are being developed for cancers with this feature. However, information on its frequency and genomic structure in non-NSCLC solid tumors from real-world clinical practice remains limited. In this study, we analyzed next-generation sequencing data from 579 patients with non-NSCLC solid tumors. *MTAP* deletion was uncommon but consistently occurred together with loss of neighboring tumor suppressor genes at chromosome 9p21, forming a spatially clustered deletion pattern centered on the 9p21 locus. This finding was observed across several cancer types, including sarcoma, pancreatic cancer, and urothelial carcinoma. In addition, all *MTAP*-deleted tumors showed low mutation burden and stable microsatellite status. These results provide real-world genomic evidence supporting *MTAP* deletion as a distinct molecular subset of solid tumors and highlight the value of comprehensive genomic profiling for identifying patients who may benefit from emerging *MTAP*-targeted therapies.

## 1. Introduction

The methylthioadenosine phosphorylase (*MTAP*) gene, located on chromosome 9p21.3 adjacent to *CDKN2A* and *CDKN2B*, encodes an enzyme essential for the methionine and adenine salvage pathways [1]. Owing to this close genomic proximity, homozygous *MTAP* deletion frequently occurs as a collateral event in tumors with 9p21 loss, resulting in the concurrent inactivation of *CDKN2A/B*, key regulators of the RB–p53 tumor suppressor network [1,2,3].

*MTAP* deficiency has been reported in approximately 10–15% of human cancers in large pan-cancer genomic analyses, although its prevalence varies substantially across tumor types and analytical platforms [4,5]. Higher frequencies have been described in selected malignancies such as pancreatic and biliary tract cancers, while more modest rates are observed in specific subtypes of lung cancer. MTAP recycles methylthioadenosine (MTA) into methionine, thereby preserving S-adenosylmethionine (SAM) pools and maintaining cellular methylation balance [6]. Loss of this enzyme leads to MTA accumulation, which competitively inhibits protein arginine methyltransferase 5 (PRMT5)—a critical mediator of histone and non-histone arginine methylation involved in transcription and RNA splicing [6,7,8]. This partial suppression of PRMT5 creates a unique synthetic lethal vulnerability, as *MTAP*-deleted tumor cells become dependent on residual PRMT5 activity for survival [9,10,11].

Building upon this vulnerability, novel MTA-cooperative PRMT5 inhibitors such as AMG 193 and MRTX1719 have demonstrated tumor-selective efficacy in preclinical and early-phase clinical studies of *MTAP*-deleted cancers [2,9].

Despite these promising advances, the clinical and molecular landscape of *MTAP*-deleted tumors remains poorly defined in real-world settings, particularly outside of non-small cell lung cancer (NSCLC), where most genomic data have been derived. To address this gap, we investigated the genomic features, co-alteration patterns, and clinicopathologic characteristics of 14 patients with *MTAP*-deleted, non-NSCLC solid tumors identified through next-generation sequencing (NGS) at our institution, aiming to provide real-world molecular evidence and clinical context for *MTAP*-deleted solid tumors in a Korean population.

## 2. Materials and Methods

### 2.1. Data Acquisition and Patient Selection

We retrospectively reviewed 579 patients who underwent next-generation sequencing (NGS) using the Oncomine Comprehensive Assay (OCA) platform (Thermo Fisher Scientific, Waltham, MA, USA) at Samsung Medical Center (data cutoff: 24 September 2025). Clinical and molecular data were extracted from the institutional sequencing database.

The OCA platform was implemented to enable comprehensive profiling of genomic alterations including *MTAP*, *CDKN2A*, and *CDKN2B*, which are recurrently altered in solid tumors and have emerging clinical relevance. Given that these tumor suppressors genes are frequently affected by copy-number loss and focal deletions, and were not sufficiently captured by previously utilized platforms, the OCA platform was selected to ensure robust detection of these loci and accurate characterization of their genomic status.

### 2.2. Somatic Mutation Landscape Visualization

Each column represents an individual patient with *MTAP* deletion, and each row corresponds to a recurrently altered gene identified across the cohort. The heatmap depicts the broader somatic mutation and copy-number landscape observed in *MTAP*-deleted tumors, extending beyond the 9p21 locus.

### 2.3. Genomic Co-Deletion Analysis

Copy-number variations (CNVs) were calculated from normalized read depth using a panel-optimized algorithm integrated within the validated clinical NGS pipeline. Read depth values were normalized for GC content and overall sequencing coverage across samples. Log_2_ copy-number ratios were derived relative to a pooled reference, and gene-level copy-number status was determined using predefined thresholds (e.g., log_2_ ratio ≤ −0.5 for copy-number loss and ≤−1.0 for homozygous deletion). Each gene was categorized as “deleted” or “neutral” based on predefined log_2_ copy-number thresholds. To assess genomic co-occurrence, binary CNV matrices were constructed for *MTAP*, *CDKN2A*, and *CDKN2B*. Intersection structures were visualized using UpSet plots (ComplexUpset, v1.3.3), and pairwise statistical associations were evaluated using two-sided Fisher’s exact tests with Haldane–Anscombe correction to stabilize odds ratio estimates. Genome-wide deletion frequencies were further mapped along chromosome 9 to localize focal 9p21.3 loss, and distance-dependent co-deletion decay from *MTAP* was quantified using linear regression and Spearman correlation.

### 2.4. Statistical Analysis

All statistical analyses were performed using R (version 4.3.2). Categorical variables were compared using Fisher’s exact test or chi-square test, as appropriate, and odds ratios (ORs) with 95% confidence intervals (CIs) were calculated for binary comparisons. Continuous variables were summarized as median with interquartile range (IQR) or median with range (min–max), as appropriate, and compared using the Wilcoxon rank-sum test. All tests were two-sided, and *p* values < 0.05 were considered statistically significant.

## 3. Results

### 3.1. Patient Characteristics

**Figure 1:**
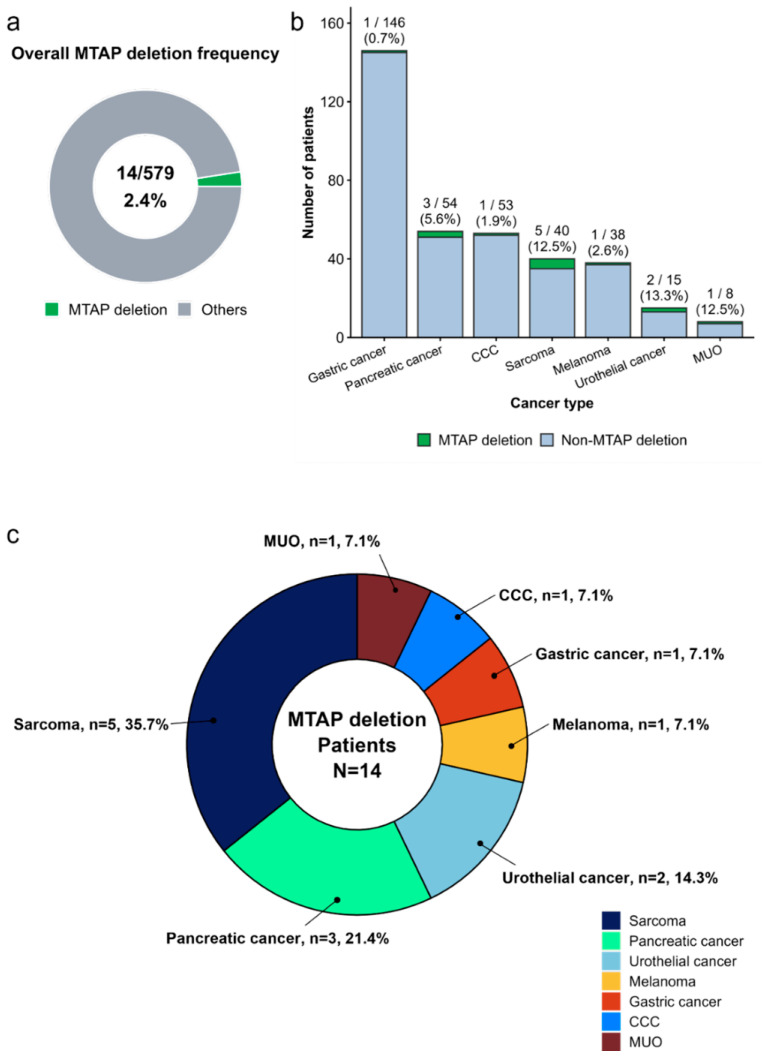
Prevalence and genomic landscape of *MTAP* deletion across cancer types. (**a**) Overall prevalence of *MTAP* deletion in the study cohort. *MTAP* deletion was detected in a minority of patients (2.4%), as shown by the small green segment of the donut plot. (**b**) Distribution of *MTAP* deletion across individual cancer types. Bars indicate the total number of sequenced patients per cancer type, with *MTAP*-deleted cases highlighted in green. The absolute count and percentage of *MTAP*-deleted tumors are annotated above each bar. (**c**) Distribution of primary cancer types among patients with *MTAP* deletion (*n* = 14). The donut chart illustrates the relative proportion of each cancer type within the *MTAP*-deleted subgroup, showing that sarcoma comprises the largest fraction, followed by pancreatic cancer and urothelial cancer, with smaller proportions of gastric cancer, melanoma, CCC, and malignancy of unknown origin (MUO).

Among 579 patients who underwent NGS-based molecular profiling, *MTAP* deletion was identified in 14 cases (2.4%; 95% CI: 1.3–4.0%). As shown in Figure 1a, *MTAP* deletion represented a small minority of the overall cohort. The frequency of *MTAP* deletion varied substantially across cancer types (Figure 1b), occurring in gastric cancer (1/146, 0.68%; 95% CI: 0.02–3.78%), pancreatic cancer (3/54, 5.56%; 95% CI: 1.16–15.39%), cholangiocarcinoma (CCC) (1/53, 1.89%; 95% CI: 0.05–10.04%), sarcoma (5/40, 12.50%; 95% CI: 4.19–26.80%), melanoma (1/38, 2.63%; 95% CI: 0.07–13.81%), urothelial carcinoma (2/15, 13.33%; 95% CI: 1.66–40.46%), and malignancy of unknown origin (MUO) (1/8, 12.50%; 95% CI: 0.32–52.65%). The median age at diagnosis in the MTAP-deleted cohort was 70 years (interquartile range [IQR], 60–75), and nine patients (64%) were male. Compared with MTAP wild-type tumors (median 63 years, IQR 55–69), patients with MTAP deletion showed a trend toward older age; however, this difference did not reach statistical significance (Wilcoxon rank-sum test, *p* = 0.069). There was no significant difference in sex distribution between patients with and without MTAP deletion (Fisher’s exact test, *p* = 0.79; odds ratio [OR] 1.23, 95% CI 0.36–4.72).

All *MTAP*-deleted tumors were characterized by low tumor mutational burden (median 3.77 mut/Mb; range 1.9–9.4) and were microsatellite stable (MSS). Homologous recombination deficiency (HRD) was detected in three patients (21%). Collectively, these features suggest reduced tumor immunogenicity based on genomic surrogates, although direct immune microenvironment profiling was not performed in this study. The distribution of cancer types within the *MTAP*-deleted cohort is depicted in Figure 1c. Sarcoma and pancreatic cancer were the most commonly represented diagnoses, together accounting for more than half of *MTAP*-deleted cases. The remaining tumors were dispersed across gastric cancer, melanoma, CCC, urothelial carcinoma, and MUO, suggesting that *MTAP* deletion is not limited to a specific histologic subtype, but rather arises across a heterogeneous spectrum of solid tumors.

### 3.2. Co-Occurrence Analysis of MTAP Deletion with CDKN2A and CDKN2B

**Figure 2:**
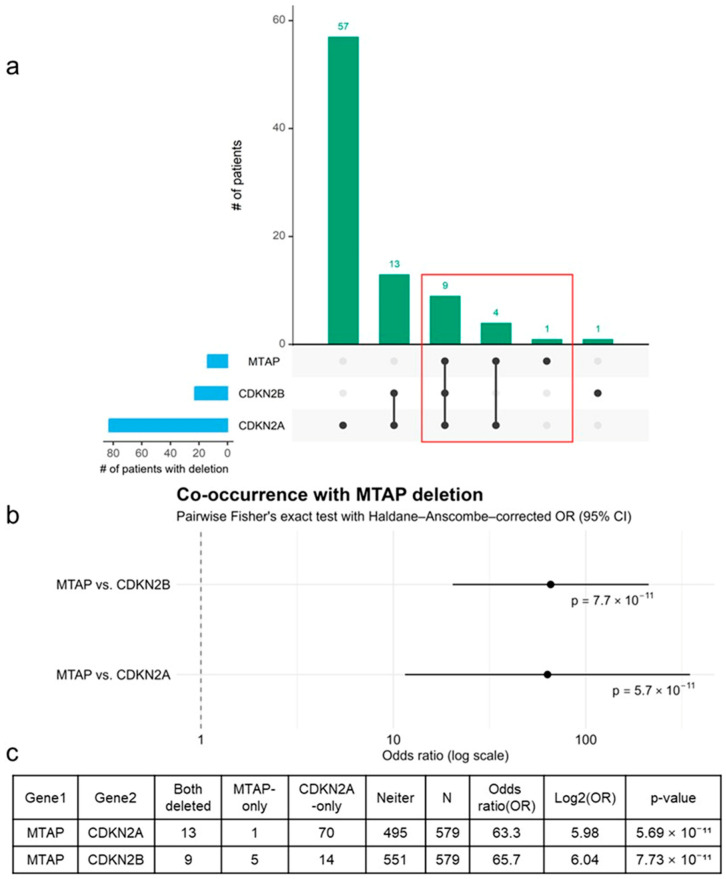
Co-occurrence analysis of *MTAP* deletion with *CDKN2A* and *CDKN2B*. (**a**) UpSet plot visualizing overlap of copy-number deletions across *MTAP*, *CDKN2A*, and *CDKN2B*, highlighting a dominant triple-deletion cluster. (**b**) Forest plot showing pairwise Fisher’s exact test results with Haldane–Anscombe-corrected odds ratios (OR > 60, *p* < 10^−10^). (**c**) Contingency tables summarizing the 2 × 2 counts for each gene pair. Together, these analyses reveal a concerted structural loss across the 9p21 tumor-suppressor locus rather than isolated *MTAP* deletions.

To determine whether *MTAP* deletion occurs as an isolated event or as part of a coordinated loss within the 9p21.3 locus, we performed a systematic co-occurrence analysis across the 579-tumor NGS cohort (Figure 2).

An UpSet plot (Figure 2a) visualized the intersection structure of copy-number deletions among *MTAP*, *CDKN2A*, and *CDKN2B*. Visualization of the intersection structure revealed that 13 of 14 *MTAP*-deleted tumors (92.9%) exhibited concurrent loss of *CDKN2A*, while 9 cases (64.3%) showed additional co-deletion of *CDKN2B*, forming a triple intersection (*MTAP* + *CDKN2A* + *CDKN2B*). Importantly, all *CDKN2B* co-deletions occurred within the subset already harboring *CDKN2A* loss, whereas *MTAP*-only deletions were exceedingly rare (1 of 14, 7.1%). This hierarchical pattern—anchored by a consistent *MTAP–CDKN2A* core deletion with occasional extension toward *CDKN2B*—suggests a single focal structural event across 9p21.3, rather than multiple independent chromosomal breaks. In essence, the deletion typically encompasses *MTAP* and *CDKN2A* as its central core and, in some tumors, expands distally to include *CDKN2B*, delineating the canonical *MTAP–CDKN2A/B* co-deletion signature.

Pairwise statistical testing using Fisher’s exact test with Haldane–Anscombe correction (Figure 2b) demonstrated a remarkably strong genomic association between *MTAP* and its neighboring tumor-suppressor genes. Specifically, *MTAP* loss was strongly correlated with *CDKN2A* (odds ratio = 63.3; *p* = 5.7 × 10^−11^) and *CDKN2B* (odds ratio = 65.7; *p* = 7.7 × 10^−11^). These extremely high odds ratios quantitatively confirm that *MTAP* deletion almost invariably co-occurs with the loss of *CDKN2A* and *CDKN2B*, reinforcing the hierarchical relationship visualized in the UpSet plot and supporting the concept of a shared focal deletion architecture across the 9p21.3 locus.

The underlying 2 × 2 contingency tables (Figure 2c) further illustrate the distribution of co-deletion events, showing that nearly all *MTAP*-deleted cases fall within the “both deleted” quadrant for *CDKN2A* and *CDKN2B*, whereas single-gene losses are rare outliers. This consistent enrichment—observed across intersection mapping, pairwise statistics, and direct count visualization—supports the presence of a focal, structurally linked deletion at chromosome 9p21.3 rather than independent chromosomal breaks.

Taken together, these findings indicate that *MTAP* deletion is not a sporadic event but a hallmark component of a concerted structural loss encompassing *CDKN2A* and *CDKN2B*. The coherence across multiple analytical layers reinforces the interpretation that deletions involving MTAP frequently extend to neighboring CDKN2A and CDKN2B within the 9p21 locus, consistent with a spatially clustered deletion pattern.

### 3.3. 9p21-Centered Deletion Pattern and Distance-Dependent Co-Deletion

**Figure 3:**
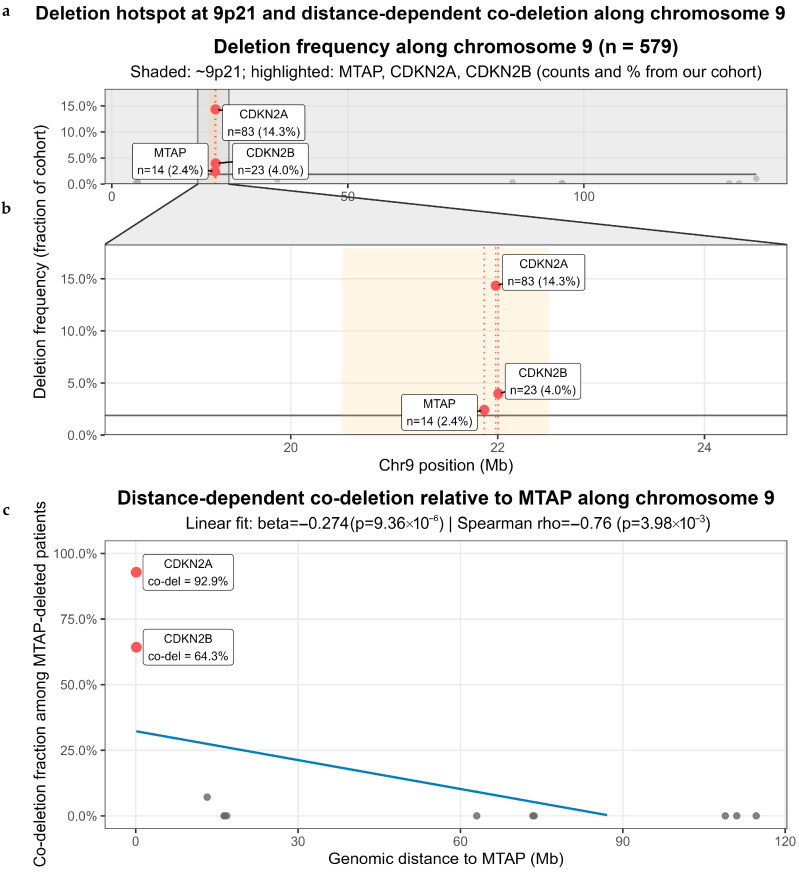
Deletion hotspot at 9p21 and distance-dependent co-deletion along chromosome 9. (**a**) Genome-wide deletion frequency along chromosome 9 in the full cohort (*n* = 579) demonstrating a peak in deletion frequency at the 9p21 locus. Cohort-level deletion counts are shown for *MTAP* (*n* = 14; 2.4%), *CDKN2A* (*n* = 83; 14.3%), and *CDKN2B* (*n* = 23; 4.0%). (**b**) Expanded view of the 9p21 region illustrating the close genomic proximity of *MTAP*, *CDKN2A*, and *CDKN2B* and overlapping deletion frequency peaks within this interval. (**c**) Among *MTAP*-deleted tumors (*n* = 14), the fraction of chromosome 9 genes co-deleted with *MTAP* decreases with increasing genomic distance from the *MTAP* locus (linear slope β = −0.274, *p* = 9.36 × 10^−6^; Spearman ρ = −0.76, *p* = 3.98 × 10^−3^), demonstrating a distance-dependent pattern of deletion clustering.

To further examine the genomic distribution underlying the observed co-occurrence of *MTAP* with neighboring tumor suppressor genes, we profiled copy-number deletion frequency along chromosome 9 in the full cohort (*n* = 579). As shown in Figure 3a, deletion events were not uniformly distributed but demonstrated a prominent peak at the 9p21 locus, where *MTAP*, *CDKN2A*, and *CDKN2B* reside. In the overall cohort, deletions were identified in 14 patients (2.4%) for *MTAP*, 83 patients (14.3%) for *CDKN2A*, and 23 patients (4.0%) for *CDKN2B*, indicating that this region represents the highest local deletion burden along chromosome 9.

A magnified view of the 9p21 region (Figure 3b) illustrates the close genomic proximity of *MTAP*, *CDKN2A*, and *CDKN2B*, with overlapping peaks in deletion frequency. *CDKN2A* exhibited the highest deletion frequency, while *MTAP* and *CDKN2B* followed a similar spatial pattern. These findings suggest that deletions involving *MTAP* frequently extend to adjacent genes within the 9p21 interval, consistent with spatial clustering in this genomic region. We next restricted the analysis to *MTAP*-deleted tumors (*n* = 14) to evaluate the spatial extent of co-deletion along chromosome 9. As shown in Figure 3c, the fraction of genes co-deleted with *MTAP* decreased progressively with increasing genomic distance from the *MTAP* locus. Linear regression analysis demonstrated a negative slope (β = −0.274, *p* = 9.36 × 10^−6^), and Spearman correlation confirmed a significant inverse association (ρ = −0.76, *p* = 3.98 × 10^−3^). Notably, genes immediately adjacent to *MTAP*, including *CDKN2A*, exhibited the highest co-deletion rates (92.9%), followed by *CDKN2B* (64.3%), supporting a distance-dependent pattern of deletion clustering.

Together, these chromosome-wide distribution profiles and distance-dependent analyses indicate that *MTAP* deletion occurs within a localized deletion hotspot centered at 9p21.3, providing genomic context for the strong statistical co-occurrence observed in Figure 2. However, given the resolution limitations of panel-based CNV profiling and the limited number of *MTAP*-deleted cases (*n* = 14), the precise structural architecture of the deletion—including the possibility of broader arm-level 9p loss—cannot be definitively determined. Accordingly, these findings should be interpreted as supportive of a 9p21-centered deletion pattern rather than as direct evidence of a uniformly focal structural event. These distance-dependent analyses should therefore be considered exploratory.

### 3.4. Mutational Landscape and Co-Deletion Context of MTAP-Deleted Tumors

**Figure 4:**
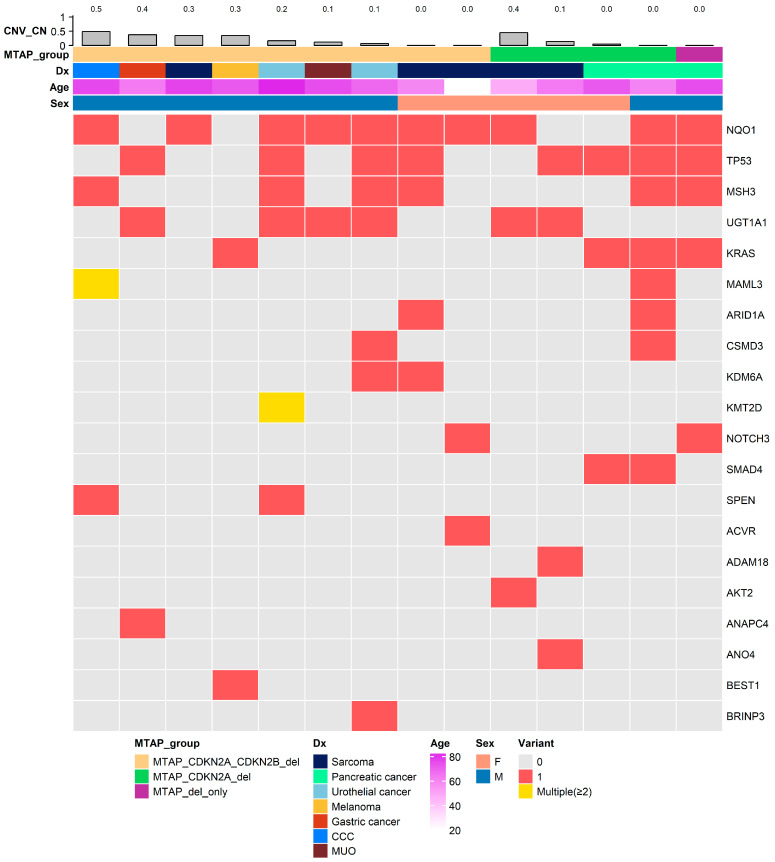
Mutational landscape of MTAP-deleted tumors (*n* = 14). Heatmap illustrating the genomic alterations observed in tumors harboring MTAP deletion. Each column represents an individual patient, and each row corresponds to a recurrently altered gene identified within this subset. Red squares indicate the presence of variant (variant = 1), yellow indicates multiple alterations (≥2 variants), and gray indicates no detected alteration (variant = 0). Top annotations display copy-number reduction magnitude (CNV_CN), MTAP co-deletion subgroup (MTAP-only, MTAP + CDKN2A-del, or MTAP + CDKN2A/B-del), primary tumor diagnosis (Dx), age at diagnosis, and sex. CNV_CN reflects gene-level copy-number reduction as derived from panel-based normalized read depth analysis. Consistent with co-occurrence and chromosome-wide analyses (Figure 2 and Figure 3), most MTAP-deleted tumors demonstrate concurrent loss of CDKN2A and/or CDKN2B. Beyond the 9p21 locus, recurrent alterations in lineage-associated driver genes (e.g., TP53, KRAS, ARID1A, KMT2D, NOTCH3) highlight the heterogeneous mutational backgrounds across tumor types. This figure contextualizes MTAP deletion within a spatially clustered 9p21 deletion pattern embedded in diverse tumor-specific genomic environments.

To further contextualize the 9p21-centered deletion pattern within the broader genomic background, we examined the mutational and copy-number landscape of the 14 patients harboring MTAP deletion. Each column in Figure 4 represents an individual patient, and each row corresponds to a recurrently altered gene identified within this subset. Top-layer annotations indicate the degree of copy-number reduction (*CNV_CN*), *MTAP* co-deletion subgroup (classified as *MTAP-only*, *MTAP* + *CDKN2A-del*, or *MTAP* + *CDKN2A/B-del*), tumor type, age, and sex, providing an integrated molecular–clinical overview.

Consistent with the co-occurrence and chromosome-wide distribution analyses presented in Figure 2 and Figure 3, the majority of MTAP-deleted tumors simultaneously exhibited CDKN2A and/or CDKN2B loss. This pattern visually supports a spatially clustered deletion architecture at the 9p21 locus; however, given the resolution limitations of panel-based CNV profiling, the precise structural extent of these deletions cannot be definitively determined.

Beyond the 9p21 region, multiple additional driver genes—including TP53, KRAS, ARID1A, KMT2D, and NOTCH3—were recurrently altered, reflecting lineage-specific oncogenic contexts, particularly among sarcoma and pancreatic cancer cases. This heterogeneity underscores that, while MTAP deletion occurs within a recurrently affected genomic interval, the broader mutational background varies substantially according to tumor lineage.

Taken together, these findings delineate a genomically coherent yet biologically heterogeneous subset of MTAP-deleted tumors. Rather than representing isolated MTAP loss events, these tumors frequently involve coordinated deletion of neighboring 9p21 tumor suppressor genes within a spatially clustered genomic region, embedded within diverse lineage-specific mutational environments.

To explore co-alteration patterns and characterize the genomic landscape associated with *MTAP* loss, we profiled somatic mutations in 14 patients harboring *MTAP* deletion. The top 20 recurrently altered genes were selected based on mutation frequency across this subset. Each column represents an individual patient, and each row denotes a gene-level alteration. Copy-number values (CNV_CN) are visualized as a barplot at the top annotation, reflecting the magnitude of copy-number reduction for each sample. Co-deletion patterns involving *CDKN2A* and *CDKN2B* are highlighted through the *MTAP*_group annotation, allowing direct visualization of 9p21 focal loss patterns. Additional clinical metadata, including diagnosis category (Dx), age, and sex, are displayed as top annotations. Overall, this heatmap depicts the heterogeneous yet coordinated genomic context surrounding *MTAP* deletion and provides visual support for co-occurring alterations within the 9p21 locus.

## 4. Discussion

This study provides institutional real-world data on the prevalence (14/579, 2.4%; 95% CI, 1.3–4.0%) and genomic features of *MTAP* deletion across non-NSCLC solid tumors in a Korean population. In large pan-cancer datasets, MTAP genomic alterations have been reported in approximately 9–15% of advanced malignancies, with substantial variation across tumor types [12,13]. For example, in the dataset by Sokol et al., MTAP alterations were observed in 9.3% of cases overall, with markedly higher frequencies in selected tumor types [14]. The lower prevalence observed in our cohort compared with pan-cancer estimates likely reflects differences in tumor-type distribution and methodological factors inherent to panel-based CNV detection.

Within the *MTAP*-deleted cohort, high co-deletion rates of *CDKN2A* (92.9%) and *CDKN2B* (64.3%) were observed. In addition, a distance-dependent decay pattern was identified along chromosome 9, suggesting spatial clustering of deletions within the 9p21.3 region rather than entirely independent random loss events. Although panel-based CNV data do not permit definitive discrimination between focal and broader arm-level alterations, the observed co-deletion pattern is consistent with the canonical 9p21.3 deletion signature described in large-scale genomic datasets [4,5].

Notably, isolated *MTAP* deletion without concurrent *CDKN2A* loss was uncommon (1/14, 7.1%), supporting the notion that *MTAP* loss frequently occurs within a broader 9p21 deletion context [15]. However, given the limited number of *MTAP*-deleted cases, these structural inferences should be interpreted cautiously.

Recent pan-cancer analyses have emphasized *MTAP* deletion has been proposed as a prognostic biomarker in prior large-scale studies and a therapeutically actionable alteration [3,4]. *MTAP* plays a central role in the methionine and adenine salvage pathways, sustaining SAM-dependent methylation homeostasis [1]. Loss of *MTAP* results in accumulation of methylthioadenosine (MTA), a metabolite that partially inhibits PRMT5 and alters downstream epigenetic regulation. In the context of the canonical 9p21 co-deletion observed in our cohort, this metabolic consequence frequently arises alongside *CDKN2A/B* loss, indicating that metabolic rewiring and RB–p53 pathway disruption commonly occur in parallel within the same 9p21 deletion context [14]. As a result, *MTAP*-deficient tumors acquire metabolic dependencies that position the PRMT5–MAT2A axis as a compelling therapeutic target [1,2,13].

Preclinical studies have shown that *MTAP*-deficient cancer cells are selectively sensitive to PRMT5 catalytic inhibition, MTA-cooperative PRMT5 inhibitors, and MAT2A blockade [2,9,13]. These insights have catalyzed the development of multiple therapeutic modalities, including (i) MTA-cooperative PRMT5 inhibitors (e.g., AMG 193, MRTX1719) [2,9], (ii) MAT2A inhibitors (e.g., AG-270) [16], and (iii) SAM-competitive PRMT5 inhibitors (e.g., JNJ-64619178) [17].

Early clinical translation of PRMT5 inhibition was demonstrated in the first-in-human phase 1 trial of PF-06939999, which showed preliminary antitumor activity and a manageable hematologic toxicity profile [18]. More recently, the *MTAP*-cooperative PRMT5 inhibitor AMG 193 has shown encouraging signals in an ongoing adaptive phase 1/1b/2 study (NCT05094336), including durable partial responses across several *MTAP*-deleted tumor types such as pancreatic and squamous NSCLC, and substantial tumor shrinkage in rare malignancies [2]. These early findings support the therapeutic feasibility of selectively targeting MTA-bound PRMT5 in *MTAP*-null cancers.

In addition to monotherapy approaches, emerging studies are increasingly exploring rational combinatorial strategies that leverage synthetic lethal interactions within specific genomic contexts [19,20]. For example, combining the *MTA*-cooperative PRMT5 inhibitor BMS-986504 with *KRAS* inhibitors has demonstrated potent antitumor activity in *MTAP*-deleted, *KRAS*-mutant pancreatic cancer models, highlighting the potential for pathway-directed synergy [19]. Together with early-phase clinical trials specifically enrolling *MTAP*-deleted patient cohorts, these developments mark a meaningful shift toward precision-medicine strategies tailored to metabolically defined cancers [21].

Beyond metabolic consequences, *MTAP* deletion has been linked to broad transcriptional, epigenetic, and immune reprogramming. Experimental and transcriptomic analyses have suggested that *MTAP*-deficient tumors may exhibit reduced interferon-γ signaling, impaired antigen presentation, and decreased CD8^+^ T-cell infiltration—features that have been described as consistent with a relatively less immunogenic tumor microenvironment [22,23]. However, direct assessment of the tumor immune microenvironment was not available in the present dataset; therefore, immune-related interpretations remain inferential and warrant further validation in studies incorporating direct immune profiling.

Emerging preclinical and early clinical data suggest that PRMT5 inhibition may modulate immune-related pathways, providing a rationale for combination strategies aimed at enhancing antitumor immunity [24]. Consistent with this concept, data from the 2025 ASCO Annual Meeting have explored epigenetic modulation via PRMT inhibition potential to reprogram the relatively less immunogenic state, and early clinical activity of the *MTAP*-cooperative PRMT5 inhibitor CTS2190 have reported enhanced immunogenicity and objective responses even in PD-(L)1-resistant tumors [24].

Across multiple large-scale genomic datasets, including Japanese nationwide comprehensive genomic profiling and TCGA analyses, *MTAP*-deleted tumors exhibit substantial prevalence and consistently poorer overall survival than *MTAP*–wild-type counterparts [4]. Although prior pan-cancer analyses have suggested an association between MTAP deletion and adverse survival outcomes, outcome analyses were not performed in the present cohort due to the limited number of MTAP-deleted cases [25,26]. Accordingly, the findings of this study should be interpreted primarily as genomic characterization rather than prognostic validation. Building on these pan-cancer observations, our study provides complementary real-world evidence by characterizing *MTAP* deletion within gastrointestinal and rare solid tumors in a Korean population, a tumor spectrum that has been underrepresented in global datasets.

Despite the frequent co-deletion of the 9p21 tumor-suppressor cluster, clinical outcomes in our cohort were markedly heterogeneous, reinforcing the biological complexity described above [27]. This variability suggests that *MTAP* deletion alone does not uniformly determine prognosis or therapeutic sensitivity; rather, its phenotypic consequences likely depend on lineage-specific tumor biology, co-occurring genomic alterations, and the extent of neighboring gene loss [28,29]. These findings highlight the importance of tumor-type-specific considerations when applying *MTAP*-based synthetic lethal strategies in clinical practice [6,30].

The major strength of this study lies in the detailed characterization of the 9p21.3 co-deletion pattern within a clinically annotated real-world cohort. Although the small sample size limits definitive prognostic or predictive conclusions, and given the limited number of MTAP-deleted cases (*n* = 14), the study was underpowered to detect modest subgroup differences, effect size estimates should be interpreted cautiously. Accordingly, lineage-specific observations in this study should be considered hypothesis-generating rather than definitive. Furthermore, because this study was designed as an NGS-based genomic analysis and *MTAP* immunohistochemical evaluation was not systematically performed, functional validation at the protein level was not available, which may further limit interpretation of the biological and clinical implications. Nevertheless, our findings provide meaningful real-world confirmation of the canonical 9p21.3 signature and underscore the clinical relevance of *MTAP* loss in gastrointestinal and rare tumors. Collectively, these findings establish an important foundation for future lineage-specific studies aimed at refining the prognostic and therapeutic implications of *MTAP* deletion and guiding the development of precision-oncology strategies tailored to *MTAP*-deficient cancers. Given the availability of emerging *MTAP*-directed therapies, routine genomic testing may facilitate the identification of eligible patients, particularly in tumor types with higher prevalence such as sarcoma, pancreatic cancer, and urothelial carcinoma.

## 5. Conclusions

In our cohort, *MTAP* deletion was detected in 2.4% of solid tumors and occurred most frequently in sarcoma, pancreatic cancer, and urothelial carcinoma. Although rare, this alteration showed a consistent 9p21.3 co-deletion pattern and genomic features characterized by low tumor mutational burden and microsatellite stability. While these findings validate the structural genomic architecture of *MTAP* deletion in a real-world setting, the limited sample size precludes definitive conclusions regarding its clinical outcomes or therapeutic implications. Collectively, these findings identify *MTAP* deletion as a small but biologically and potentially clinically relevant molecular subset, providing a structural genomic foundation for future lineage-specific validation and prospective therapeutic investigation.

## Data Availability

The data presented in this study are available from the corresponding author upon reasonable request.
